# Open Access to essential health care information

**DOI:** 10.1186/1477-7819-2-42

**Published:** 2004-12-02

**Authors:** Christabel EL Stokes, Manoj Pandey

**Affiliations:** 1 BioMed Central, 34-42 Cleveland Street, London, UK; 2 Regional Cancer Centre, Thiruvananthapuram, Kerala, India

## Abstract

Open Access publishing is a valuable resource for the synthesis and distribution of essential health care information. This article discusses the potential benefits of Open Access, specifically in terms of Low and Middle Income (LAMI) countries in which there is currently a lack of informed health care providers – mainly a consequence of poor availability to information. We propose that without copyright restrictions, Open Access facilitates distribution of the most relevant research and health care information. Furthermore, we suggest that the technology and infrastructure that has been put in place for Open Access could be used to publish download-able manuals, guides or basic handbooks created by healthcare providers in LAMI countries.

## 

'Essential healthcare information' is the basic information required by primary health care workers to perform their role within the community. This basic information would be most useful if it is informed by relevant research, produced locally, and made available in the local language. The potential benefits of Open Access in terms of access to the research literature in general, and to research from low- and middle-income (LAMI) countries in particular, have been well described elsewhere.

We would like to introduce a new dimension into this debate: Open Access has an untapped potential to enhance the synthesis and distribution of essential healthcare knowledge. Open Access, as opposed to free access, allows readers the right to use the article without restriction. Local publishers can therefore filter, reproduce and distribute the most relevant research and healthcare information from any and all Open Access journals. In essence, they can create journals focused on local issues based on content from a variety of journals. These "local journals" can be circulated in print – a medium that remains essential in countries with limited computer and Internet access. To our knowledge, this has yet to be done, although we are hoping someone will exploit this opportunity soon.

In the future, we imagine the technology and infrastructure that has been developed for Open Access could be used to publish download-able manuals, guides or basic handbooks created by healthcare providers in these countries. These free resources could then be accessed worldwide and, where necessary, reproduced within local communities in the optimal medium. In an "author-pays" Open Access model the charges would be standard and could be covered by a national government organization or charity.

Open Access will increase the availability of research and, in doing so, stimulate researchers in LAMI countries to develop their own research and practices. With research published in the Open Access medium it also becomes possible for producers of healthcare materials to optimize the use of research produced from their own and other countries. Thus, Open Access will optimize the distribution of local healthcare information, with potential benefits worldwide.

## Competing Interests

CELS is an employee of BioMed Central, an Open Access publisher that is funded through article-processing charges levied on accepted manuscripts. CELS receives a fixed salary, which is unaffected by the amount of money received by BioMed Central from article-processing charges.

MP is the Editor-in-Chief of *World Journal of Surgical Oncology*, an Independent journal published by BioMed Central.

